# Effect of TiO_2_ Abutment Coatings on Peri-Implant Soft Tissue Behavior: A Systematic Review of In Vivo Studies

**DOI:** 10.1155/2024/9079673

**Published:** 2024-03-19

**Authors:** Nagat Areid, Faleh Abushahba, Sini Riivari, Timo Närhi

**Affiliations:** ^1^Department of Prosthetic Dentistry and Stomatognathic Physiology, Institute of Dentistry, University of Turku, Turku FI-20520, Finland; ^2^Department of Biomaterials Science and Turku Clinical Biomaterial Center-TCBC, Institute of Dentistry, University of Turku, Turku FI-20520, Finland; ^3^Department of Restorative Dentistry and Periodontology, Faculty of Dentistry, Libyan International Medical University (LIMU), Benghazi 339P+62Q, Libya; ^4^Wellbeing Services County of South-West Finland, Turku FI-20521, Finland

## Abstract

Establishing a proper soft tissue adhesion around the implant abutment is essential to prevent microbial invasion, inhibit epithelial downgrowth, and obtain an optimal healing process. This systematic review aims to evaluate the real potential of TiO_2_ coating on the behavior of peri-implant soft tissue health and maintenance. A specific aim was to evaluate clinically and histologically the effect of TiO_2_ abutment coating on epithelial and connective tissue attachment. Electronic database searches were conducted from 1990 to 2023 in MEDLINE/PubMed and the Web of Science databases. In total, 15 out of 485 publications were included. Eight studies involved humans, and seven were animal studies. Exposure time ranges from 2 days to 5 years. The peri-implant soft tissue evaluations included clinical assessment (plaque index (PI), peri-implant probing pocket depth (PPD), and bleeding on probing (BoP)), histological as well as histomorphometric analysis. The Office of Health Assessment and Translation (OHAT) Risk of Bias Rating Tool for Human and Animal Studies was used to evaluate the overall quality of the studies included in the review. The results showed some variation but remained within acceptable limits. Within the limitations of this systematic review, the present findings suggest that TiO_2_ coatings seem to influence soft tissue healing. TiO_2_-coated abutments with a roughness value between 0.2 and 0.5 *μ*m enhance soft tissue health. Sol-gel-derived TiO_2_ coatings induced better soft tissue attachment than noncoated machined abutment surfaces. The anodized titanium abutments demonstrate comparable clinical and histological outcomes to conventional machined abutments. However, there was variation among the included studies concerning TiO_2_ coating characteristics and the measured outcomes used to evaluate the soft tissue response, and therefore, quantitative analysis was not feasible. Long-term in vivo studies with standardized soft tissue analysis and coating surface parameters are necessary before a definitive conclusion can be drawn. OSF Registration No.: 10.17605/OSF.IO/E5RQV.

## 1. Introduction

The use of implants in dentistry is a routine treatment modality nowadays. Implant abutment materials and their surface characteristics are considered crucial factors in improving the clinical performance of dental implants [1, 2]. The interaction of the implant abutment surfaces with soft tissue is guided mainly by their surface wettability, roughness, and topography, which have been considered the key influencing factors that affect the initial cell responses at the cell–material interface and ultimately promote a bond with the surrounding tissues [2]. It has been shown that abutments with good surface wettability have the potential to enhance epithelial and connective tissue contact with the abutment surface [3]. Machined titanium (Ti) surfaces have been used clinically for implant abutments to impede bacterial adhesion and biofilm formation. However, the surface modification to the implant surface at the transmucosal level has been shown to facilitate peri-implant soft tissue attachment without promoting bacterial colonization and reduce the risk of bone resorption [2, 4, 5].

Various surface modification techniques have been developed to optimize osseointegration at the bone tissue–implant interface. Moderately rough surfaces (Sa = 1–2 *μ*m; with Sa being the arithmetic mean of the absolute values of the surface departures from the mean plane) at the bone level have shown good osteogenic properties and more bone formation than minimally rough surfaces (Sa = 0.5–1 *μ*m) [6, 7]. In comparison, at the soft tissue interface, the formation of epithelium and connective tissue seal between the implant abutment and the surrounding soft tissue is essential to hinder bacterial infiltration, inhibit epithelial downgrowth, and obtain optimal healing [8–10]. Therefore, surface modification techniques, such as grit-blasting, acid etching, Ti plasma spraying, electrochemical anodic oxidation, and laser treatment, have enhanced surface bioactivity and created surface structures with different topographies for guided tissue regeneration [11–14].

The junctional epithelium (JE) attaches to the tooth surface via basal lamina and hemidesmosomes along the epithelia–tooth interface, while the peri-implant epithelium attachment is weaker and displays slower cell proliferation to the implant surface. In addition, the internal basal lamina and hemidesmosomes have been found only at the apical region of the epithelia–implant interface [15, 16]. This difference in epithelial attachment indicates that the peri-implant epithelial tissue has a lower functional sealing capacity than the JE around natural teeth [10]. In addition, the parallel orientation of the collagen fibers to the implant surface weakens the defense mechanism around the implants, making them more susceptible to epithelial downgrowth and bacterial invasion when compared with natural dentition [17, 18]. Recent studies, however, have shown the presence of fibers oriented perpendicular/oblique to the modified transmucosal abutment surface [19, 20]. Therefore, it might be expected that implant surface modification could influence cell attachment and healing process at the Ti–tissue interface.

Different surface modifications of the implant transmucosal component have been studied previously to ensure proper soft tissue attachment and stability. TiO_2_ coating is one example of surface modifications that have shown promising potential to improve soft tissue attachment on implant surfaces [21]. Various techniques have been used to obtain bioactive TiO_2_ coatings on implant surfaces. These include plasma spraying [22], anodic oxidation [23], a sol–gel coating method [24], and hydrothermal (HT) treatment [25]. Despite developing various TiO_2_ surface modification techniques to produce bioactive coatings on medical devices, their widespread clinical adoption remains challenging. Coating techniques need to be biocompatible, simple, reproducible, and financially viable for industrial-scale application while accommodating complex shapes and topographies.

Modifying the Ti implant surface at the nanoscale level can alter surface chemistry and topography, influencing the implant surface interaction with ions, proteins, and cells [26]. These interactions can enhance molecular and cellular activities and promote tissue healing at the Ti–tissue interface [27, 28]. Several in vitro studies have demonstrated that nanoporous TiO_2_ coatings enhanced gingival cell response [5, 21, 29, 30]. It has been shown that nanostructured TiO_2_ coatings with smooth surface topography and good wettability promote favorable fibroblast and epithelial cell responses compared to machined surfaces which ultimately affects the rate and quality of new tissue formation [31]. However, in vitro studies cannot truly represent the biological process of oral implant and abutment integration. Furthermore, there is limited in vivo data on the impact of these surface properties on the soft tissue–implant interface. Therefore, this systematic review aims to investigate the in vivo potential of TiO_2_ abutment coatings on soft tissue health and maintenance. A specific aim was to evaluate the effect of TiO_2_ abutment coating on epithelial and connective tissue attachment.

## 2. Materials and Methods

The studies selected in the present systematic review were screened according to Preferred Reporting Items for Systematic Reviews and Meta-Analysis (PRISMA) guidelines [32]. The research question was: “What is the effect of TiO_2_ coated abutment on soft tissue-implant interface?”. The question was established based on the PICOS (Population–Intervention–Comparison–Outcomes and Setting) strategy:

- Population: soft tissue implant interface.
- Intervention: abutments with TiO_2_ coatings.
- Comparison: noncoated machined abutment.
- Outcomes: soft tissue response with a qualitative and/or quantitative evaluation.
- Setting: in vivo studies.

The review protocol was registered in Open Science Framework (OSF) Registries. The PRISMA checklist for this study is reported in the electronic supplementary material (*Supplementary 1*).

### 2.1. Eligibility Criteria

Original research papers, randomized control trials, nonrandomized observational studies, and prospective and retrospective studies were eligible for this review. Solely in vivo studies evaluating the effect of TiO_2_-coated abutments on soft tissue–implant interface response were considered; this included studies of human and animal models. Studies with a surface modification other than TiO_2_ coating were not included. Also, Studies addressing only the interface between bone and Ti were eliminated. Only the soft tissue aspect was considered if a selected paper reported soft and bone tissue results. In addition, only articles written in English were selected.

### 2.2. Information Sources and Search Strategy

The systematic literature search was conducted using the electronic database searches of (MEDLINE/PubMed and the Web of Science). The search filters applied included peer-reviewed articles written in English and published between 1990 and 2023. Keywords related to TiO_2_-coated implant/abutments were combined with keywords related to soft tissues with AND/OR as Boolean operators, as shown in Table 1. The search process was extended by filtering the reference lists of the selected studies to identify any additional studies related to the topic.

### 2.3. Study Selection and Data Extraction

The selection process of the electronic search results was carried out by excluding the duplicates. Two independent researchers (FA and NA) screened the title and abstract. Then, the same reviewers assessed the full texts of the studies meeting the criteria and those with insufficient data in the title and abstract. Irrelevant studies were eliminated, and the fourth author (TN) was contacted for clarification in case of disagreement. The inter-rater reliability was evaluated using kappa coefficients. Eligible articles were stored in an electronic full-text version. Two reviewers (NA and SR) extracted data independently and inserted it into two Excel spreadsheets for animal and clinical studies designed for this purpose. The following variables were extracted from each study: the author's name and year of publication, study model, study design, TiO_2_ coating techniques, number of implant/abutment, treatment and control interventions, coating thickness, soft tissue analysis, and trial duration.

### 2.4. Risk of Bias in Individual Studies

The quality assessment of the selected studies was performed using the Office of Health Assessment and Translation (OHAT) Risk of Bias (ROB) Rating Tool for Human and Animal Studies [33]. The OHAT ROB assessment tool is a parallel approach that assesses the quality and ROB in human and animal studies. The ROB assessment was conducted independently for human and animal studies. The analysis was done at the study level and was carried out by two independent reviewers (FA and NA). The OHAT ROB criteria are organized under six ROB domains: selection, confounding, performance, attrition/exclusion, detection, and selective reporting ROB, with sets of criteria for each one separately (*n* = 11). These criteria assess randomization, allocation concealment, experimental conditions, blinding, incomplete data, exposure characterization, outcome assessment, reporting, and other biases related to the methodological structure. Each domain was rated as “definitely low,” “probably low,” “probably high,” or “definitely high” ROB, depending on the descriptions given for each item in the selected studies. Any discrepancies between the judgments of the two reviewers were clarified by discussion.

## 3. Results

### 3.1. Study Selection

**Figure 1:**
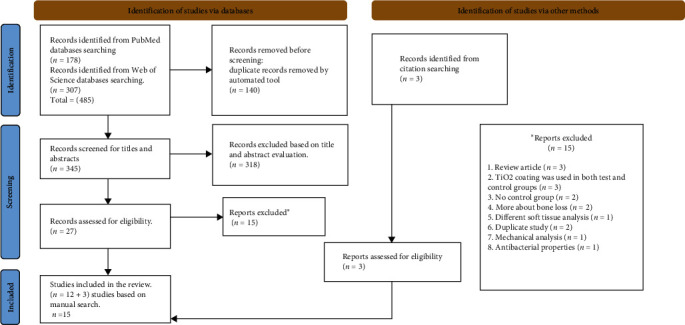
PRISMA flow diagram of the study selection procedure. ( ^*∗*^) indicates reports excluded box which explains the reasons for excluding articles.

The PRISMA flow diagram reporting, screening, and selection of studies is presented in Figure 1. From the initial search, 485 publications were identified (178 articles from PubMed and 307 articles from Web of Science). After the initial title and abstract screening, 27 papers were selected for full-text evaluation based on the inclusion and exclusion criteria. In addition, three publications were added manually. A total of 15 studies were included in this systematic review [34–48]. Kappa values for title and full-text overall agreement were 0.76 and 0.82, respectively, indicating a good agreement.

### 3.2. Quality Assessment-Risk of BIAS in Individual Studies

Table 2 illustrates the scores for each criterion of the included studies. The results showed some deviation but were considered acceptable. Performance bias was considered “Probably High” in Welander et al. [34], Rossi et al. [36], Ungersböck et al. [40], Glauser et al. [47], Schupbach and Glauser [48], Fukayo et al. [37], Paldan et al. [38], and Areva et al. [39], because insufficient information provided about blinding to study group during the study. Detection reporting bias was considered at “Probably High” risk in Glauser et al. [47], Schupbach and Glauser [48], Fukayo et al. [37], Paldan et al. [38], Areva et al. [39], Raes et al. [43], and Dib-Zaitum et al. [45] because of indirect evidence indicating lack of adequate blinding of the clinician during outcome assessment. Attrition bias was considered “Probably High” by Paldan et al. [38], and Ungersböck et al. [40], due to insufficient information about animal loss. Selection bias was “Probably High” in Welander et al. [34], Rossi et al. [36], Fukayo et al. [37], Ungersböck et al. [40], and Glauser et al. [47] because of insufficient information about allocation to study groups. In contrast, it was “Definitely High” in Schupbach and Glauser [48] because there was no randomization to the type of abutment each patient received.

### 3.3. Study Characteristics

After full-text screening, 15 studies were selected for detailed analysis and data extraction [34–48]. In all included studies, commercially pure (cp) Ti or a Ti6Al4V alloy was used for the implant. A machined or polished surface was defined as the control surface. The study design varied in terms of study model and duration time. Eight selected studies involved humans [41–48], and seven were animal studies [34–40], including rats, dogs, pigs, and sheep. Exposure time ranges from 2 days to 5 years. The implant geometry also varied between the studies, from discs and cylinders to screws. Various TiO_2_ coating techniques were used, including anodized techniques, Sol–gel derived TiO_2_ coatings, TiO_2_-blasted techniques, laser treatment, and oxidized techniques. The peri-implant soft tissue evaluation included clinical assessment (plaque index (PI), peri-implant probing pocket depth (PPD), and bleeding on probing (BoP)), histological as well as histomorphometric analysis. Due to the differences in animal models and TiO_2_ coating techniques used among studies, results were compiled in subgroups based on the study design: animal and human studies. The selected animal and human studies and their main characteristics are given in Tables 3 and 4, respectively.

#### 3.3.1. Animal Studies

In this group, a total of seven studies, distributed as follows: rats (3), dogs (2), pigs (1), and sheep (1) were included. These animal experiments were all designed to investigate soft tissue response to TiO_2_-coated implant surfaces using histological and histomorphometric analysis. The studies included differed in terms of the soft tissue type used. Four studies used oral mucosal tissue [34–37], two used dorsal subcutaneous tissue [38, 39], and one used muscle tissue [40]. Also, the follow-up time varied between 2 days and 4 months (Table 3).

Areva et al. [39] investigated the soft tissue response to sol-gel-derived TiO_2_ coating (thickness: 380 nm; Ra = 0.88 nm; with Ra being the arithmetic average of the absolute values of the profile heights) on a cp Ti implant inserted subcutaneously in a rat model. After 2 days of implantation, the connective tissue was attached to the titania-coated surfaces, whereas the noncoated control Ti showed no evidence of connective tissue attachment. After 7 days, the coated surfaces were surrounded by connective tissue components; meanwhile, a few collagen fibers and fibroblasts were seen on the control surfaces. On day 12, the histological analysis showed that the connective tissue was in immediate contact with the coated Ti. In contrast, a clear gap and a fibrous capsule formed between the connective tissue and the noncoated Ti controls.

Moreover, their follow-up study evaluated the strength of soft tissue attachment to sol–gel derived TiO_2_ coatings (thickness: 380 nm; Sa = 0.26 *μ*m) with different aging time, on cp Ti using a rat model [38]. At all-time points (3, 11 and 90 days), the coated surfaces showed close contact with the surrounding soft tissues with no clear connective tissue capsule, while the connective tissue capsule was visible around the noncoated group. The strength of soft tissue attachment was measured by pullout force. The rupture forces were higher for coated than noncoated Ti implants, whereas sol aging time does not influence soft tissue attachment. Scanning electron microscope (SEM) evaluation, carried out immediately after the pullout measurement, showed connective tissue remnants on the coated implants. In contrast, no connective tissue was detected on the noncoated implants. This indicated that with coated implants, the tissue rupture happened within the connective tissue layer rather than at the tissue–implant interface, as observed in the control implants [38].

The same research group compared the peri-implant soft tissue attachment between TiO_2_-coated (thickness: 380 nm; Sa = 0.26 *μ*m), and noncoated cp Ti surfaces at the transmucosal part of ITI® implants in a beagle dog model for 8 weeks [36]. Histological analysis showed mild inflammatory reactions in peri-implant connective tissues around the coated implants. Moreover, the JE appeared in intimate contact with the coated implant surface. In contrast, a thin gap was observed between the noncoated implant surface and the JE, leading to minor cell adhesion. In addition, 45% of the control implants displayed a total detachment of JE compared to 22% of the coated implants. Transmission electron microscopic evaluation demonstrated dense plaques of hemidesmosomes on the JE cell membrane facing the coated implants [36].

Alternatively, Ungersböck et al. [40] studied the effect of Ti plate implants with different surface treatments on the soft tissue response at the interface. The Ti plates were placed on the tibia under the leg muscles using the sheep model. They found that Ti anodized plates with a coarse surface (Ra = 0.76 ± 0.1 *μ*m) and Ti milled (Ra = 0.91 ± 0.1 *μ*m) showed good soft tissue adhesion with small connective tissue fibers, which ruptured when lifting off the tissue layer after 12 weeks of implantation. In contrast, a nonadherent and thick soft tissue capsule was observed for fine anodized Ti plates (Ra = 0.44 ± 0.1 *μ*m) with parallel orientated fibers. On the other hand, Susin et al. [35] compared the peri-implant soft tissue response around anodized abutment (thickness:153 ± 5 nm; Sa = 0.1 *μ*m) using an intraoral mini pig model. No statistically significant differences in the inflammation scores and epithelium length were observed between the control and test groups at any point in healing time. Mucosal height was significantly higher at 3 weeks in favor of the control group, but this difference was not observed at 6 and 13 weeks. The soft tissue healing around Ti abutments with either a turned or a moderately rough TiO-blast surface was studied by Welander et al. [34] using a mongrel dog model. After 4 months of healing, the connective tissue in contact with the test abutment showed a higher density of collagen and a lower number of fibroblasts than that at the turned control abutment.

Similarly, Fukayo et al. [37] evaluated the gingival connective tissue responses toward nanosecond-pulsed laser-treated Ti implants (with micro-scale roughened oxide layers) inserted into the extracted sockets of rat maxillary molars. The histological analysis demonstrated better attachment of gingival connective tissue to Laser-Ti implants after 3 and 6 weeks of implantation compared to nontreated Ti implants. Moreover, Polarized light microscopy showed rod-like attachments of gingival collagen fibers running perpendicular to the Laser-Ti implant surfaces. In contrast, there were no detectable attachments with the gingival connective tissue along the control implant surface.

#### 3.3.2. Clinical Studies

In this group, eight clinical studies were included, six randomized [41–46] and one nonrandomized trial [47], while the study design of one clinical trial was not reported [48]. These clinical trials were designed to evaluate the effect of TiO_2_-coated abutments on peri-implant soft tissue healing. Three studies used mini-implants [41, 47, 48], two used healing or temporary abutments, and a short follow-up period ranged from 3 days to 14 weeks [44, 45]. The other three studies used permanent abutments connected to fixed or removable prostheses with a more extended follow-up period of 1.5–5 years [42, 43, 46]. The soft tissue evaluation was expressed as clinical assessment, histological, and histomorphometric analysis.

Glauser et al. [47] produced microporous TiO_2_ coating on experimental one-piece mini implants using microarc oxidation. A total of 12 implants with an oxidized, a machined, or an acid-etched surface were inserted in five patients and harvested following a transmucosal healing period of 8 weeks. The histomorphometric analysis of the soft tissue barrier demonstrated less epithelial downgrowth and longer connective tissue seal at TiO_2_-modified implants than machined implants. At the same time, the collagen fibers of the connective tissue seal were run parallel to the implant surfaces. The same group conducted a follow-up study using the same material to investigate the structural and ultrastructural features between transmucosal Ti implants and their surrounding tissues interface using light and transmission electron microscopy [48]. After an 8-week healing period, no substantial differences were observed for the epithelial interfaces concerning all evaluated implant surfaces. In contrast, depending on the implant texture, significant morphologic differences were noted in how the implants interface with connective tissue. Implants with oxidized surfaces revealed the attached connective tissue with collagenous fibrils functionally oriented toward the implant surface [48].

Moreover, Wennerberg et al. [41] prepared nanoporous TiO_2_ coating (thickness: 380 nm; Ra = 0.88 nm) on experimental microimplants utilizing a sol–gel coating method. Thirty experimental microimplants were placed in 15 patients to compare the histologic features of nanoporous TiO_2_-coated against noncoated surfaces. At the time of implant retrieval (14 weeks), the TiO_2_-coated implants demonstrated healthier and firmer soft tissue attachment than the noncoated implant. The mean percentage of the oral mucosa (epithelium and connective tissue) in contact with the implant transmucosal part was significantly higher for the coated group (72%) than that of the control group (48%). However, no difference was observed in the number of inflammatory cells and fibroblasts, the sulcus depth, or the height of the marginal gingiva between the two implant surfaces.

Göthberg et al. [42], in a 5-year follow-up study, analyzed the behavior of soft tissue next to machined and TiUnite® abutments in 50 partially edentulous patients (with good oral hygiene) treated with a three-unit fixed prosthesis using delayed or immediate loading procedure. No raw periodontal parameters were available except for bone level. However, based on their observations, the authors rejected the hypothesis that a moderately rough (oxidized) abutment facilitates a soft tissue seal from the surrounding oral environment. Both machined and oxidized abutments revealed soft tissue regression. In addition, similar levels of BoP and PPD were found between machined and oxidized abutments.

Similarly, Raes et al. [43] compared the clinical performance of the minimally machined surface and moderately rough (TiUnite®) abutment surface (Sa = 1.1 *μ*m) in patients with a history of severe periodontitis. Patients were either fully or partially edentulous with teeth in the opposing jaw having remaining pockets and treated with full-arch fixed bridge or overdenture. After 5 years of follow-up, the TiUnite® abutments showed increased bone loss, PPD, clinical attachment loss, and BoP compared to the machined abutment. However, the differences between both surfaces were statistically insignificant. The cumulative survival rate was 97.6% for machined and 100% for oxidized implants/abutments. The authors concluded that in patients with a history of severe periodontitis, minimally rough implants/abutments showed more favorable clinical outcomes than moderately rough implants/abutments surfaces.

Alternatively, Farrag and Khamis [46] evaluated the peri-implant soft tissue health around anodized and nonanodized abutments connected to osseointegrated implants in 30 patients. Each patient received two abutments: one anodized abutment (experimental group) and one nonanodized abutment (control group). The peri-implant soft tissue was evaluated using PPD, soft tissue recession, modified sulcus bleeding index, modified PI, and modified gingival index. Throughout the evaluation period (18 months), no statistically significant differences were observed between anodized and nonanodized abutments in relation to peri-implant soft tissue health.

Comparable results have been reported by Hall et al. [44], who studied the effect of a nanostructured anodized abutment surface (thickness: 100 nm; Ra = 0.2 *μ*m) on healing and soft tissue health. A total of 35 patients received a pair of anodized (test) and machined (control) abutments. The abutments placed at the time of implant insertion were replaced after 6 weeks by definitive abutments with the same surface properties and followed up after 6 months and 2 years. Anodized abutments showed significantly lower soft tissue bleeding upon abutment removal at a 6-week follow-up and a greater height of keratinized mucosa throughout the 2-year follow-up, indicating better soft tissue outcomes than control abutments. However, no significant differences were detected between the anodized and machined groups concerning the PPD, PI, and signs of inflammation (redness and swelling index). Furthermore, significant differences in gene expression markers were observed between the test and control groups, indicating differences in soft tissue healing and remodeling.

In contrast, Dib-Zaitum et al. [45] analyzed clinically and histologically the response of the peri-implant soft tissue to transgingival abutment with or without surface treatment. Ten patients with edentulous maxilla received four implants placed in the area of the first and second molars on both sides and connected with either anodized or machined abutments for 8 weeks. The machined abutments showed better performance at both the epithelial and connective levels, with an epithelium height of 1.52 mm and a connective tissue height of 2.3 mm, than the anodized abutment with epithelium and connective heights of 2.02 mm and 1.74 mm, respectively. The anodized abutments tended to be in contact with a denser connective tissue. However, no significant differences were observed in the dimensions of the biological width, the density of the collagen fibers, and the number of inflammatory cells between the two abutment surfaces.

## 4. Discussion

It is well-known that establishing a proper soft tissue adhesion at the abutment-soft tissue interface is crucial for successful dental implants. However, forming this bond between the implant abutments and the surrounding soft tissue is not easily achieved. It has been found that the wound healing process around dental implants is characterized by a high proinflammatory state, resulting in fibrous capsule formation that hinders the direct attachment of adjacent soft tissue to the abutment surface [49]. This soft tissue seal around the implant abutment is essential to prevent microbial invasion, inhibit epithelial downgrowth, and obtain an optimal healing process [50].

Although increased implant surface roughness has been indicated to enhance bone-to-implant contact, it encourages biofilm formation, which may affect peri-implant soft tissue health and maintenance [51, 52]. Therefore, more knowledge is needed about the influence of surface topography on the development and maintenance of the soft tissue barrier. Some studies state that surface roughness values smaller than the “critical threshold” of 0.2 *μ*m are often preferred for abutments [53]. However, there is good evidence that modifying the transmucosal implant surface may enhance peri-implant soft tissue attachment [33, 54–57]. In the early stage, moderately rough-surfaced abutment could aid in soft tissue integration [56, 57]. This was supported by the findings of the present systematic review, where better soft tissue adhesion with good connective tissue seal and higher density of collagen fibers were observed at the TiO_2_-coated abutments than at the control-turned abutment surface [34, 40]. Perpendicular gingival collagen fibers were also observed on the Laser-Ti implant surface at 6 weeks, resulting in better connective tissue attachment [37]. Consistent with these findings, recent studies indicated that, at the early stage of healing, modified abutment surfaces encourage the formation of perpendicular collagen fibers attachment to the abutment surface [19, 20]. This positive effect appears less efficient over an extended period without any negative consequences, although some studies observed increased biofilm formation on modified surfaces [51, 52].

Gingival attachment to implant surfaces can be enhanced by altering surface properties, such as modifying surface topography, wettability, or bioactivity. Nanoporous TiO_2_ coatings are one example of surface modifications frequently investigated to improve soft tissue attachment on implant surfaces. Previous in vitro studies have shown that nanostructured TiO_2_-coating improves surface wettability and modified surface topography by creating a smooth and nanoporous structure [58, 59]. The higher wettability, which represents the hydrophilic behavior of the nanostructure TiO_2_ surface, promoted protein adsorption, encouraged cell adhesion and proliferation, and consequently enhanced connective tissue regeneration [29, 30]. Furthermore, the author's previous in vitro review has shown that TiO_2_ coatings with smooth surface topography and good wettability promote fibroblast and epithelial cell response compared with machined surfaces [31]. However, the outcomes of the present systematic review did not consistently align with these findings. Based on the animal studies included in this review, nanoporous sol-gel-derived TiO_2_ coated implants were able to reduce epithelial downgrowth and facilitate direct soft tissue attachment compared to noncoated machined implants [36, 38, 39]. The mechanism underlying soft tissue attachment to sol-gel-derived TiO_2_ coating remains unclear. However, it appears that nanoporous TiO_2_ thin films attract proteins like fibronectin, facilitating cell attachment [39]. Other factors influencing soft tissue reactions include macrophage preference for rough surfaces [60, 61]. Smooth sol-gel-derived TiO_2_-modified surfaces lack signals for macrophages, preventing interleukin-1 (IL-1) formation and thereby reducing capsule formation. Moreover, capsule formation typically occurs around implants with low-energy surfaces. Cells do not adhere to a low-energy surface but instead bind to each other, often leading to the formation of a capsule [61]. The presence of specific high-energy TiOH-groups in titania gel, containing anatase and rutile structures, induces calcium phosphate formation and enhances biomolecule adhesion, leading to better tissue integration [62–64]. Anatase and rutile structures on these surfaces allow for closer attachment of physiological fluids, proteins, and soft tissues compared to amorphous Ti structures, facilitating enhanced adhesion and spreading of connective tissue cells [36, 38, 39, 64].

The findings of the previous animal experiments were supported by a clinical study that evaluated the soft tissue attachment to sol–gel TiO_2_ surface-modified implants [41]. According to Wennerberg et al. [41], the nanoporous TiO_2_-coated abutments could promote soft tissue attachment on abutment surfaces. Therefore, it has a potential clinical benefit in improved healing and reduced bone resorption. In addition, a comparable beneficial effect of nanotopography was also demonstrated with microarc oxidized TiO_2_ coatings [47, 48]. The oxidized implant revealed less epithelial down growth and a longer connective tissue seal than the machined implant [47, 48]. It also influenced the alignment of collagen fibers adjacent to the implant, as indicated by functionally oriented fibers, which may impede the downgrowth of the JE. Meanwhile, with the smooth machined surface, most fibers were parallel to the implant surface [48]. This may indicate that the abutment surface texture may affect the orientation of collagen fibers of the connective tissue at the abutment surface. However, the precise mechanism influencing the orientation of fibers concerning smooth and rough surfaces remains unidentified.

On the contrary, the longer term prospective clinical studies (5 years) included in this review reported different results [42, 43]. Based on Göthberg et al. [42] and Raes et al. [43], the oxidized (TiUnite®) abutments reported a less favorable outcome than the machined Ti surface. While only patients with good oral hygiene were included in the Göthberg et al. [42] study, all the patients in the study conducted by Raes et al. [43] had a history of severe periodontitis. The authors concluded that in patients with a history of severe periodontitis, minimally rough (machined) implants/abutments showed more favorable clinical outcomes than moderately rough (oxidized) implants/abutments surfaces. This difference might be attributed to patients with a history of severe periodontal disease having inadequate oral hygiene procedures [65], and the moderately rough abutment surface may require extra effort in their maintenance. However, the differences between both surfaces were statistically insignificant [42, 43].

Anodic oxidation is another surface modification technique used to obtain a bioactive Ti oxide layer on the Ti surface. This technique alters the surface topography and surface chemistry, which are essential for the biological response [23]. Based on this in vivo systematic review, there were no significant differences in soft tissue response between anodized and nonanodized Ti abutments [35, 45, 46]. This finding suggests that the anodized modified surface does not interfere with peri-implant soft tissue health.

On the other hand, Hall et al. [44] study demonstrated better soft tissue outcomes for anodized abutment compared with conventional machined abutment with a similar surface roughness value of approximately 0.2 *μ*m. However, no significant effect was observed on bacterial colonization and proteolytic activity between test and control abutment [44]. The discrepancy between the studies may be attributed to factors other than the type of abutment surfaces, such as the implant site, implant maintenance, smoking habits, and medical conditions of patients. These findings seem to agree with the results of a recent systematic review analyzing peri-implant tissue behavior around Ti abutment surface modifications [66]. According to Canullo et al. [55, 66], surface modifications of Ti abutments did not appear to have a detrimental impact on peri-implant soft tissue in the short term. The results of the present systematic review indicated that TiO_2_ coatings appear to enhance soft tissue attachment, which could positively influence clinical outcomes.

In this systematic review, strict criteria for selecting studies have been implemented to minimize variability, and several studies were excluded due to their failure to report the specific outcomes of interest for this review. The studies' quality was evaluated using the OHAT ROB rating tool for both human and animal studies. All studies were acceptable according to the criteria set by this assessment.

A quantitative analysis (meta-analysis) was not possible in this review due to variations in the methods and measured outcomes among the studies included. Consequently, only qualitative analysis was conducted. Another limitation of this review is that the TiO_2_ coatings in the included studies were produced using different techniques, including the anodized method [35, 40, 44–46], oxidized method (TiUnite®) [42, 43], sol-gel-derived TiO_2_ coatings [36, 38, 39, 41], microarc oxidation [47, 48], TiOblast technique [34], and laser treatment [37]. Also, there was inconsistency in reported surface characterization and coating properties. Few studies provided detailed information about the surface characteristics. However, these data primarily focused on coating thickness and average roughness. Moreover, the follow-up period is widely different between the included studies, ranging from 2 to 5 years, making comparisons more difficult. Furthermore, certain studies present findings based on a limited sample size, and therefore, a clear conclusion on the optimal TiO_2_ coating properties to improve the soft tissue-abutment interface cannot be drawn. Further, in vivo studies with standardized methods for evaluating soft-tissue attachment and standardized surface parameters of TiO_2_ coatings are recommended to evaluate the soft tissue-abutment interface.

## 5. Conclusion

Within the limitations of this in vivo systematic review, the present findings suggest that TiO_2_ coatings seem to promote soft tissue healing. TiO_2_-coated abutments with a roughness value between 0.2 and 0.5 *μ*m enhance soft tissue adhesion. Coatings produced by MAO and sol-gel-derived TiO_2_ coatings induced better soft tissue attachment than noncoated machined abutment surfaces. The anodized Ti abutments demonstrate comparable clinical and histological outcomes to conventional machined abutments.

## Figures and Tables

**Table 1:** Search strategy and keywords.

#1	“Dental implant” or “healing abutment” or abutment ^*∗*^ or “dental abutment” or “oral implant” or “prosthetic abutment”
#2	“Titanium dioxide” or TiO_2_ ^*∗*^ or “titanium oxide” or titanium dioxide coat ^*∗*^ or titanium oxide coat ^*∗*^ or surface modif ^*∗*^ or modified surface ^*∗*^ or nanotube ^*∗*^ or nanostructure ^*∗*^ nanoporous ^*∗*^
#3	“peri-implant soft tissue” or gingiva ^*∗*^ or fibroblast ^*∗*^ or “human gingival fibroblast” or “Gingival epithelial cell” or keratinocyte ^*∗*^ or mucosa ^*∗*^ or “tissue-implant interface” or “peri-implant tissue”
#4	titanium ^*∗*^ or “Ti6Al4V” or Zircon ^*∗*^
#5	#1 and #2 and #3 and #4

( ^*∗*^) indicates variable endings of a root word.

**Table 2:** Risk of bias results for the studies using the OHAT rating tool.

Authors	Selection bias	Confounding bias	Performance bias	Attrition bias	Detection bias	Selective reporting bias
Glauser et al. [47]	−	+	−	++	−	+
Schupbach and Glauser [48]	−−	+	−	+	−	+
Wennerberg et al. [41]	++	++	+	+	+	++
Göthberg et al. [42]	++	+	++	+	++	+
Raes et al. [43]	++	++	+	+	−	++
Hall et al. [44]	++	++	+	++	+	++
Dib-Zaitum et al. [45]	++	+	++	++	−	++
Farrag and Khamis [46]	++	++	+	++	+	+
Ungersböck et al. [40]	−	+	−	−	+	++
Susin et al. [35]	++	++	+	++	++	++
Fukayo et al. [37]	−	−	−	+	−	++
Rossi et al. [36]	−	++	−	+	+	+
Areva et al. [39]	+	+	−	+	−	+
Paladan et al. [38]	+	+	−	−	−	+
Welander et al. [34]	−	−	−	+	+	+

*Note*: “++”: definitely low risk of bias; “+”: probably low risk of bias; “–”: probably high risk of bias; and “– –”: definitely high risk of bias.

**Table 3:** Included animal studies with their major characteristics.

Authors	Animal model	No. of implants	Implant type	Ti grade	TiO_2_ coating technique	Coating thickness/surface roughness	Soft tissue analysis	Time of exposure
Areva et al. [39]	Rat: 86	(i) 96 (48 test, 48 ctr) (ii) 36 (24 test, 12 ctr)	(i) Titanium discs (ii) Cylindrical implants (5 mm × 7 mm)	CpTi Grade 2	Sol–gel coating	Coating thickness = 380 nm Ra (TiO_2_ coating) = 0.88 nm	SEM histological histomorphometric analyses	(i) 2, 7 days (ii) 3, 12 days

Fukayo et al. [37]	27 rat	27 (9 laser—Ti, 9 Coll/laser—Ti, 9 Ti ctr)	Cylindrical titanium implants (1 mm × 4.5 mm)	CpTi Grade 2	Nanosecond-pulsed laser treatment, oxide layer	(Diagram without associated values), The Sa value of Laser-Ti is higher than Ti ctr	Histological analyses	3 weeks 6 weeks

Paldan et al. [38]	23 rat	(i) 72 (54 test, 18 ctr) (ii) 56 (28 test, 28 ctr)	(i) Cylindrical implants (5 mm × 7 mm) (ii) Discoid implants (5 mm × 1 mm)	CpTi Grade 2	Sol–gel coating	TiO_2_-coated implants Sa = 0.255 *μ*m	Histological and histomorphometric analyses	(i) 3, 11, 90 days (ii) 14, 21 days

Rossi et al. [36]	six beagle dogs	40 (24 test, 16 ctr)	ITI straumann implants (4.1 mm × 8 mm)	CpTi Grade 4	Sol–gel coating	Coating thickness = 380 nm TiO_2_-coated implants Sa = 0.255 *μ*m	Histological and histomorphometric analyses	8 weeks

Susin et al. [35]	24 mini pigs	96 (48 test, 48 ctr)	NobelActive (3.5 mm × 10 mm) implant and multi-unit abutment 2.5 mm	Grade 5	Anodized	Coating thickness = 153 ± 5 nm anodized abutment: Sa = 0.1 *μ*m	Histological and histomorphometric analyses	3, 6, 13 weeks

Ungersböck et al. [40]	10 sheep	(8 Ti anod. Fine 7 Ti anod. medium 7 Ti anod. coarse 7 Ti milled)	Limited contact dynamic compression titanium plates	CpTi	Anodized	Ra, (*μ*m): Ti anod. Fine = 0.44 ± 0.1 Ti anod. medium = 0.32 ± 0.1 Ti anod. Coarse = 0.76 ± 0.1 Ti milled = 0.91 ± 0.1	Histological and histomorphometric analyses	3 months

Welander et al. [34]	five mongrei dogs	20 (10 test, 10 ctr)	OsseoSpeed, Astra Tech Dental (3.5 mm × 8 mm)	Grade 4	TiOblast	NA	Histological and histomorphometric analyses	4 months

Abbreviations: ctr, control; SEM, scanning electron microscope; Ti, titanium; cpTi, commercially pure titanium; anod., anodized; Ra, the arithmetic average of the absolute values of the profile heights; Sa; the arithmetic mean of the absolute values of the surface departures from the mean plane; and NA, not available.

**Table 4:** Included human studies with their characteristics.

Authors	Country	Study type	Study design	No. of patients	No. of implants	TiO_2_ coating technique	Prosthesis type	Soft tissue analysis	Exposure time
Glauser et al. [47]	Switzerland	NA	NA	5 pt	12 Screw-shaped one-piece mini-implant, 2.3 mm × 10 mm (four test four ctr four acid etched)	Micro arc oxidation oxidized and micro—porous TiO2 layer	4 mm cylindrical abutment portion in contact with the soft tissue	(i) Clinical observation (ii) Peri-implant soft tissue barrier (qualitative and quantitative scoring analysis: (iii) Histological analysis (iv) Histomorphometric analysis	8 weeks

Schupbach and Glauser [48]	Switzerland	nonrandomized	Implants placed in sequence and/or bilaterally.	5 pt	12 Screw-shaped one-piece mini-implants (Nobel Biocare AB), 2.3 mm × 10 mm. (four test four ctr four acid etched)	oxidized and micro—porous TiO2 layer	4 mm cylindrical abutment portion in contact with the soft tissue	(i) Structural and ultrastructural features of the interface between transmucosal implants and surrounding tissues (ii) Histological analysis (iii) SEM	8 weeks

Wennerberg et al. [41]	Sweden & Finland	Randomized allocation	Split mouth	15 pt	30 experimental micro implants 10 and 13 mm long, × 2.2 mm width), (15 test; 15 ctr)	Sol–gel derived TiO2 (MetAlive®, Vivoxid Ltd., Turku, Finland). Coating thickness = 380 nm Ra (TiO_2_ coating) = 0.88 nm	Abutment part 3.4 mm and 6.4 mm long	(i) Clinical investigation (ii) Histological analysis	14 weeks

Göthberg et al. [42]	Sweden	Randomized allocation	Controlled parallel	50 pt	150 Brånemark TiUnite implants (Nobel Biocare AB) (62 test; 64 ctr)	Oxidized (TiUnite)	Three-unit fixed prosthesis connected directly at implant level or via machined abutment or an oxidized abutment (TiUnite)	(i) Soft tissue height coronal from the abutment platform to the mucosal margin (ii) Plaque index (PI) (iii) Peri-implant probing pocket depth (PPD) (iv) Bleeding on probing (BoP)	2 days, 2 and 4 weeks, 3 and 6 months, and 1, 3, and 5 years.

Raes et al. [43]	Belgium	Randomized allocation	Split mouth	18 patients with a history of severe periodontitis	84 Brånemark MK III (Nobel Biocare AB) (42 test; 42 ctr)	Oxidized (TiUnite)	Full arch fixed or overdenture with machined or oxidized (TiUnite) abutments	(i) Probing pocket depth (PPD), (ii) Clinical attachment level (CAL), (iii) Bleeding on probing (BoP), (iv) Microbiological analysis	1, 3, 5 years
Hall et al.[44]	Sweden	Randomized allocation	Split mouth	35 pt	70 Brånemark Mk III or Mk IV, (TiUnite, Nobel Biocare AB) (35 test; 35 ctr)	Nanostructured anodized coating thickness = 100 nm Ra = 0.2 *μ*m	abutments with anodized or machined surface	(i) Soft tissue assessments: (ii) Bleeding on probing(BoP) (iii) Height of keratinized mucosa (iv) Redness and swelling (v) Plaque accumulation (vi) Peri-implant probing depth(PPD) (vii) Gene expression analysis by qPCR	6-week, 6-month, and 2-year

Dib-Zaitum et al. [45]	Spain	Randomized allocation	Split mouth	10 pt	40 endosseous implants (IPX−4010_Galimplant) (20 anodized, 20 machined)	Anodized	Transepithelial abutments with anodized or machined surfaces	(i) Clinical measurement (ii) Histological parameter (iii) Histomorphometric analysis	8 weeks

Farrag and khamis [46]	Egypt	Randomized allocation	Split mouth	30 pt	60 Screw bone-level implants (Dentium)(30 test; 30 ctr)	Anodized	cement-retained crowns on anodized or non-anodized abutments	(i) Clinical parameters: (ii) Peri-implant probing depth (PPD) (iii) Soft tissue recession (iv) Modified sulcus bleeding index (v) Modified plaque index (vi) Modified gingival index	At insertion (baseline), 3, 6, 12 and 18 months

Abbreviations: NA, not available; ctr, control; qPCR, quantitative polymerase chain reaction; SEM, scanning electron microscope; Ra, the arithmetic average of the absolute values of the profile heights; and pt, patients.

## Data Availability

All the data is available in the main text.

## Additional Points

*Summary Box*. *What is known*: TiO_2_ coating is a surface modification that has shown good potential to improve soft tissue attachment on implant surfaces. A recent systematic review suggested TiO_2_ coatings with smooth surface topography promote gingival cell response. This systematic review was based on in vitro studies and cannot truly represent the biological process of oral implant and abutment integration. *What this study adds*: This review aims to evaluate the in vivo potential of TiO_2_ coatings on peri-implant soft tissue health and maintenance. TiO_2_-coated abutments with a roughness value between 0.2 and 0.5 *μ*m enhance soft tissue health, which could positively influence clinical outcomes.

## Supplementary Materials

Supplementary Materials Supplementary 1 in the Supplementary Material for The PRISMA checklist for this study.
